# Regional IVIM-DWI Abnormalities in Normal-Appearing White Matter and Lesion Tissue in Relapsing–Remitting Multiple Sclerosis

**DOI:** 10.3390/jcm15145493

**Published:** 2026-07-13

**Authors:** Othman I. Alomair, Sami A. Alghamdi

**Affiliations:** Radiological Sciences Department, College of Applied Medical Sciences, King Saud University, P.O. Box 145111, Riyadh 4545, Saudi Arabia; oalomir@ksu.edu.sa

**Keywords:** relapsing–remitting multiple sclerosis, normal-appearing white matter, IVIM-DWI, diffusion-weighted imaging, white matter lesions, quantitative MRI

## Abstract

**Background/Objectives:** Normal-appearing white matter (NAWM) in relapsing–remitting multiple sclerosis (RR-MS) may contain abnormalities not visible on conventional MRI. This study evaluated whether intravoxel incoherent motion diffusion-weighted imaging (IVIM-DWI) detects regional NAWM abnormalities and differentiates non-lesional white matter from lesion tissue in RR-MS. **Methods:** This retrospective cross-sectional single-center study included 189 patients with RR-MS and 26 healthy controls. Apparent diffusion coefficient (ADC), true diffusion coefficient (D), pseudo-diffusion coefficient (D*), and perfusion fraction (f) were measured in the genu, splenium, frontal white matter, and posterior white matter. Regional MS NAWM was compared with the anatomically corresponding control white matter. Tissue-compartment analysis compared averaged control white matter, averaged MS NAWM, and lesion-averaged MS tissue. **Results:** Posterior white matter showed the strongest abnormalities, with lower ADC, D, D*, and f in RR-MS than in controls (all *p* < 0.001); ADC, D, and D* showed large effects (Cohen’s d: −0.81 to −1.07). In the splenium, ADC and D were higher, whereas D* was lower in RR-MS. After age and sex adjustment, posterior white matter ADC, D, and D* and splenial D* remained significant, whereas posterior f and splenial ADC/D were attenuated. No clear IVIM-DWI abnormality was detected in the sampled frontal white matter. Lesion-averaged MS tissue showed higher ADC, D, and D* than averaged MS NAWM and control white matter (all *p* < 0.001). **Conclusions:** Regional IVIM-DWI identified region-specific NAWM abnormalities in RR-MS, most robustly in posterior white matter, within an exploratory imaging framework. Larger matched longitudinal cohorts are required before clinical or prognostic utility can be established.

## 1. Introduction

Multiple sclerosis (MS) is a chronic inflammatory and neurodegenerative disease of the central nervous system characterized by demyelination, axonal injury, gliosis, and progressive tissue damage [1,2,3,4]. Globally, MS affects approximately 2.8 million people, corresponding to an estimated prevalence of 35.9 per 100,000 population [2]. MRI is essential for MS diagnosis and monitoring because it demonstrates lesion dissemination in space and time and enables detection of focal inflammatory demyelinating lesions [5,6,7,8]. Conventional MRI sequences, including FLAIR, T2-weighted imaging, and pre- and post-contrast T1-weighted imaging, are highly sensitive for detecting visible MS lesions and inflammatory activity [5,7]. However, conventional lesion metrics do not fully explain clinical heterogeneity or disability accumulation, reflecting the recognized clinicoradiological gap in MS [9,10]. This limitation has increased interest in advanced quantitative MRI techniques that can detect tissue abnormalities beyond visible lesions [11].

Normal-appearing white matter (NAWM) is increasingly recognized as an important tissue compartment in MS. Although NAWM appears normal on conventional MRI, it may contain subtle microstructural damage, demyelination, axonal injury, gliosis, inflammatory changes, and altered water mobility [12,13,14,15,16]. Importantly, NAWM abnormalities are not necessarily diffuse or uniform. Spatially resolved studies have shown that abnormalities may occur in regions where new lesions subsequently develop, suggesting that NAWM may represent an intermediate tissue state between healthy white matter (WM) and focal MS lesions [13,17]. Diffusion MRI studies have also demonstrated measurable NAWM alterations in RR-MS, and these alterations have been associated with brain volume, clinical disability, and regional tissue vulnerability [12,14]. These findings support the need for regional NAWM assessment rather than relying only on global averages, which may obscure localized abnormalities.

The regional distribution of NAWM abnormalities is clinically and biologically relevant. MS lesions preferentially involve specific anatomical locations, including periventricular, juxtacortical, infratentorial, spinal cord, and callosal regions [5,6,7]. The corpus callosum is particularly important because of its dense interhemispheric fiber organization and frequent involvement in demyelinating disease. Posterior WM may also be relevant to MS-related microstructural injury because of its anatomical connectivity and proximity to lesion-prone periventricular regions [12,13,14]. Therefore, evaluating anatomically distinct WM regions may help determine whether MS-related NAWM abnormalities are region-specific rather than globally distributed.

Diffusion-based techniques are widely used to characterize microstructural tissue injury in MS. The apparent diffusion coefficient (ADC) reflects overall water diffusivity, whereas diffusion tensor imaging (DTI)-derived parameters provide information on directional water diffusion and WM fiber organization; these measures have shown abnormalities in MS lesions, perilesional tissue, diffusely abnormal WM, and NAWM [12,14,15,16,18]. Other quantitative approaches, including diffusion kurtosis imaging (DKI) and multicomponent T2 relaxation, have also demonstrated spatially variable abnormalities in non-lesional WM, supporting the value of regional quantitative MRI assessment in MS [19,20]. However, ADC, DTI, DKI, and relaxation-based measures characterize selected aspects of tissue injury and do not directly separate diffusion-related from perfusion-related signal components. In this context, intravoxel incoherent motion diffusion-weighted imaging (IVIM-DWI) provides a complementary non-contrast approach that separates true molecular diffusion from pseudo-diffusion related to microvascular perfusion [21,22,23]. Within the IVIM-DWI framework, the true diffusion coefficient (D) represents molecular diffusion, the pseudo-diffusion coefficient (D*) reflects incoherent microvascular motion, and the perfusion fraction (f) represents the fractional contribution of the perfusion-related signal component. Accordingly, IVIM-DWI may provide complementary information on tissue diffusivity and microvascular perfusion, both of which are relevant to MS pathophysiology [21,22,23,24].

Prior IVIM-DWI studies in MS have shown that IVIM parameters can characterize lesion heterogeneity and differentiate lesion types, including enhancing, non-enhancing, and T1-hypointense black hole lesions [25]. IVIM-derived metrics have also demonstrated associations with disability-related measures in RR-MS, although lesion count and relapse frequency may remain stronger independent predictors of disability than IVIM parameters alone [26]. In parallel, IVIM-DWI combined with radiomics has shown potential for lesion phenotyping and clinical status prediction [27]. These studies support the biological relevance of IVIM-DWI in MS lesion assessment. However, regional IVIM-DWI abnormalities in NAWM remain insufficiently characterized, particularly when evaluated together with healthy control WM and lesion-averaged MS tissue.

A tissue-compartment framework may provide a clearer understanding of IVIM-DWI patterns in MS. Healthy control WM represents normal reference tissue, MS NAWM represents macroscopically normal but potentially altered tissue, and MS lesions represent focal pathological tissue. Integrating these compartments within the same analytical framework may help determine whether IVIM-DWI parameters differ across normal WM, non-lesional MS tissue, and focal MS lesion tissue, and whether regional NAWM abnormalities are diluted by global averaging or concentrated in specific WM regions.

Building on this rationale, the present study aimed to evaluate regional IVIM-DWI abnormalities in NAWM in patients with RR-MS compared with healthy controls and to examine IVIM-DWI patterns across three tissue compartments: healthy control WM, MS NAWM, and lesion-averaged MS tissue. We hypothesized that IVIM-DWI would reveal quantitative abnormalities in RR-MS NAWM compared with healthy control WM and would demonstrate distinct parameter profiles between non-lesional MS tissue and established MS lesion tissue. Four predefined WM regions were examined: the genu, splenium, frontal WM, and posterior WM.

## 2. Materials and Methods

### 2.1. Study Design and Participants

This retrospective cross-sectional single-center study included 189 patients with RR-MS and 26 healthy controls. The RR-MS cohort was retrospectively identified from clinical and imaging records acquired between 2019 and 2024. All MS cases recorded in the institutional Picture Archiving and Communication System (PACS) during the study period were screened, and patients were assessed for eligibility if they had undergone the standardized MS MRI protocol that included IVIM-DWI. Thus, the RR-MS cohort represents a retrospective, consecutive, complete-case sample of eligible patients with available, analyzable IVIM-DWI data, rather than a prospectively matched or convenience-selected cohort.

Patients with RR-MS were included if they had a confirmed diagnosis according to the 2017 McDonald criteria [6], available conventional MRI and IVIM-DWI, analyzable regional NAWM measurements, and lesion-averaged IVIM-DWI data. The RR-MS diagnosis was verified from clinical neurology reports based on the treating neurologist’s assessment. Patients were excluded if image quality was insufficient for ROI analysis or if essential imaging measurements were incomplete.

The RR-MS patient imaging data were acquired using a standardized MS MRI and IVIM-DWI protocol. The healthy control group served as the reference white matter (WM) cohort and was acquired under a separate approved healthy-control imaging study using the same IVIM-DWI b-value scheme as the RR-MS imaging protocol. Healthy controls were adults with structurally normal brain MRI and no known neurological or psychiatric disorders. All control MRI examinations were reviewed by a board-certified neuroradiology consultant and confirmed to be free of visible brain abnormalities. Controls were excluded if they had significant motion degradation, imaging artifacts, or incomplete quantitative MRI data. The complete-case requirement was based on the availability of conventional MRI, IVIM-DWI, and regional WM/NAWM measurements; lesion-averaged IVIM-DWI measurements were additionally required for RR-MS patients. Participant selection for the RR-MS and healthy-control cohorts is summarized in Supplementary Figure S1.

The study was conducted in accordance with the Declaration of Helsinki and was approved by the Institutional Review Board of King Saud University Medical City. The RR-MS cohort was approved under IRB No. E-23-7517, and the healthy control cohort was approved under Project No. E-25-9848. Consent was waived for the retrospective RR-MS analysis, while written informed consent had been obtained from all healthy control participants in the source healthy-control study, including permission for future research use of the imaging data.

### 2.2. MRI Acquisition

MRI examinations were performed using 1.5-T GE MRI systems (GE Healthcare, Waukesha, WI, USA) with phased-array head coils. The RR-MS MRI protocol included three-dimensional T1-weighted imaging, three-dimensional FLAIR, axial T2-weighted imaging, pre- and post-contrast T1-weighted imaging, and multi-b-value diffusion-weighted imaging for IVIM analysis, following the standardized acquisition approach used in the source RR-MS imaging cohort [25]. Healthy control IVIM-DWI data were acquired using the same b-value scheme as the RR-MS imaging protocol.

IVIM-DWI was acquired using b-values of 0, 30, 50, 70, 100, 200, 500, and 1000 s/mm^2^ in three orthogonal diffusion directions. The IVIM-DWI data were used to generate ADC, D, D*, and f maps [25,26].

### 2.3. IVIM-DWI Processing

IVIM-DWI data were processed using the IB Diffusion™ plug-in, version 21.12 (Imaging Biometrics, Elm Grove, WI, USA), integrated into OsiriX MD, version 12.0 (Pixmeo SARL, Bernex, Switzerland). A segmented IVIM model was applied using a b-value threshold of 200 s/mm^2^. The true diffusion coefficient (D) was estimated primarily from higher b-values above this threshold, whereas the pseudo-diffusion coefficient (D*) was estimated from the low-b-value signal component. The perfusion fraction (f) was calculated from the perfusion-related signal contribution, and the apparent diffusion coefficient (ADC) was generated using a mono-exponential model, as previously described [25,28]. ADC, D, and D* were expressed in ×10^−3^ mm^2^/s.

### 2.4. NAWM ROI Definition

Regional NAWM measurements were obtained from four predefined WM regions: the genu, splenium, frontal WM, and posterior WM. The genu and splenium were selected to represent the anterior and posterior corpus callosum, whereas the frontal and posterior WM ROIs were selected to sample non-callosal supratentorial WM regions. This approach allowed assessment of whether IVIM-DWI abnormalities in NAWM differed by anatomical location rather than being uniformly distributed across the sampled WM regions.

All ROIs were manually placed by an experienced neuroradiologist using ITK-SNAP software, version 3.8.0, a validated open-source tool for medical image segmentation [29]. Formal blinding to group status was not feasible because ROI placement required differentiation between RR-MS and healthy-control datasets and, in RR-MS patients, careful identification and avoidance of visible MS lesions. To reduce measurement bias, ROIs were placed using predefined anatomical locations and standardized avoidance criteria. In patients with RR-MS, ROIs were placed within normal-appearing tissue while avoiding visible MS lesions, CSF spaces, vascular structures, and imaging artifacts. In healthy controls, anatomically corresponding WM ROIs were placed in structurally normal WM and were used as the normal reference tissue.

**Figure 1:**
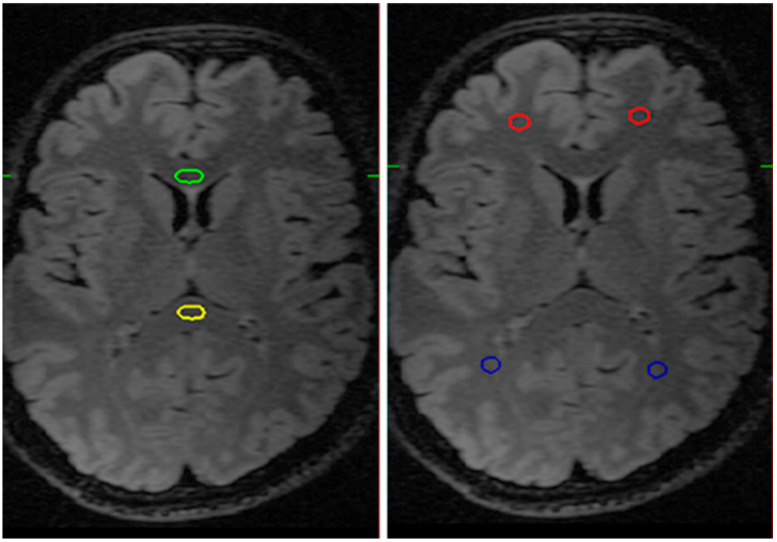
Representative placement of measurement regions on axial FLAIR images. The genu of the corpus callosum is shown in green, the splenium in yellow, bilateral frontal WM in red, and bilateral posterior WM in blue. Right- and left-sided frontal and posterior WM measurements were averaged to generate one frontal WM value and one posterior WM value for each participant. These regions were used for the extraction of ADC, D, D*, and f values. ADC = apparent diffusion coefficient; D = true diffusion coefficient; D* = pseudo-diffusion coefficient; f = perfusion fraction; ROI = region of interest; WM = white matter.

ADC, D, D*, and f values were extracted from each predefined ROI. For the bilateral frontal and posterior WM regions, right- and left-sided ROI values were averaged to generate a single frontal WM value and a single posterior WM value for each participant. The same bilateral averaging procedure was applied to patients with RR-MS and healthy controls. For the tissue-compartment analysis, an averaged MS NAWM value was calculated for each IVIM-DWI parameter by averaging the four NAWM regions within each RR-MS patient. An averaged control WM value was calculated using the same four anatomically corresponding WM regions in healthy controls. Representative ROI placement is shown in Figure 1.

### 2.5. MS Lesion IVIM Measurements

MS lesions were identified on conventional MRI sequences, including FLAIR, T2-weighted imaging, and post-contrast T1-weighted imaging. Lesion identification and ROI placement were performed by an experienced neuroradiologist. Lesion ROIs were manually delineated using ITK-SNAP software, version 3.8.0, with guidance from co-registered anatomical images, as previously described [25]. ROIs were placed within representative lesion areas while avoiding partial-volume effects, motion artifacts, and adjacent non-lesional tissue.

For each patient with RR-MS, lesion-level IVIM-DWI measurements were summarized as lesion-averaged ADC, D, D*, and f values. Lesion count was recorded for descriptive characterization of the RR-MS cohort.

### 2.6. Tissue Compartments

Three tissue compartments were defined for analysis: healthy control WM, MS NAWM, and MS lesion tissue. Healthy control WM was used as the normal reference compartment, MS NAWM represented macroscopically normal-appearing tissue in patients with RR-MS, and MS lesion tissue represented focal pathological tissue.

Regional analysis compared MS NAWM with anatomically corresponding control WM across the four predefined regions: genu, splenium, frontal WM, and posterior WM. Tissue-compartment analysis compared averaged control WM, averaged MS NAWM, and lesion-averaged MS tissue to characterize IVIM-DWI patterns across normal reference WM, macroscopically normal-appearing MS tissue, and focal MS lesion tissue.

### 2.7. Statistical Analysis

Statistical analyses were performed using IBM SPSS Statistics, version 26.0 (IBM Corp., Armonk, NY, USA). Continuous variables were summarized as mean ± SD, and categorical variables as *n* (%). Normality was assessed using the Shapiro–Wilk test. Because several IVIM-DWI parameters showed non-normal distributions, non-parametric tests were used for group comparisons.

For the primary regional analysis, IVIM-DWI parameters in MS NAWM and anatomically corresponding control WM regions were compared using the Mann–Whitney U test. Cohen’s d was calculated to quantify the magnitude and direction of between-group differences. To assess the influence of demographic imbalance between RR-MS patients and healthy controls, age- and sex-adjusted sensitivity analyses were performed using linear regression models, with each regional IVIM-DWI parameter as the dependent variable and group status, age, and sex as independent variables. False-discovery-rate adjustment was applied to the adjusted regional comparisons.

For the tissue-compartment analysis, pairwise comparisons were performed across averaged control WM, averaged MS NAWM, and lesion-averaged MS tissue. Comparisons involving healthy controls were performed using the Mann–Whitney U test, whereas comparisons between averaged MS NAWM and lesion-averaged MS tissue were performed using the Wilcoxon signed-rank test because both measurements were obtained from the same RR-MS patients.

All statistical tests were two-tailed, and *p* < 0.05 was considered statistically significant. No prospective sample-size or power calculation was performed because this was a retrospective, complete-case imaging study. Given the exploratory regional design, *p*-values were interpreted together with effect-size patterns rather than as isolated indicators of significance.

## 3. Results

**Figure 2:**
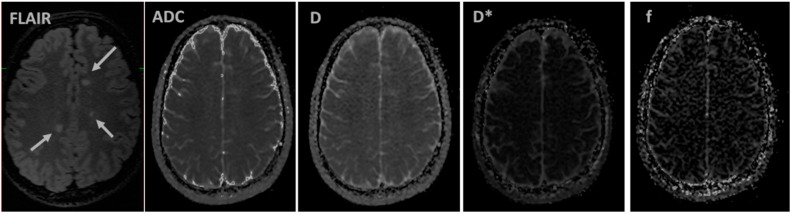
Representative axial FLAIR and IVIM-DWI parameter maps from a patient with relapsing–remitting multiple sclerosis (RR-MS). FLAIR imaging demonstrates multiple white matter lesions (arrows). Corresponding apparent diffusion coefficient (ADC), true diffusion coefficient (D), pseudo-diffusion coefficient (D*), and perfusion fraction (f) maps are shown. The figure illustrates the imaging basis for lesion identification and IVIM-DWI parameter extraction in RR-MS. ADC, D, and D* are expressed in ×10^−3^ mm^2^/s.

Figure 2 presents representative MRI findings from a patient with RR-MS, including conventional FLAIR imaging and corresponding IVIM-DWI parameter maps.

### 3.1. Participant Characteristics

The final analysis included 189 patients with RR-MS and 26 healthy controls. The RR-MS group had a mean age of 36.02 ± 9.31 years, whereas the healthy control group had a mean age of 31.73 ± 9.23 years. The RR-MS cohort included 57 males (30.2%) and 132 females (69.8%), while the healthy control group included 23 males (88.5%) and 3 females (11.5%). Among patients with RR-MS, the mean disease duration was 6.27 ± 5.18 years, the mean EDSS score was 2.25 ± 1.94, and the mean lesion count was 11.57 ± 8.19 lesions. A total of 138 patients (73.0%) were receiving DMT, whereas 51 patients (27.0%) were not receiving DMT. Participant characteristics are summarized in Table 1.

### 3.2. Regional NAWM IVIM-DWI Differences Between RR-MS Patients and Healthy Controls

Regional IVIM-DWI parameters were compared between RR-MS NAWM and anatomically corresponding control WM across the genu, splenium, frontal WM, and posterior WM (Table 2). The most pronounced abnormalities were observed in posterior WM. Compared with controls, patients with RR-MS showed significantly lower posterior WM ADC (0.766 ± 0.102 vs. 0.862 ± 0.054 × 10^−3^ mm^2^/s, *p* < 0.001, d = −0.99), D (0.729 ± 0.104 vs. 0.810 ± 0.050 × 10^−3^ mm^2^/s, *p* < 0.001, d = −0.81), and D* (0.894 ± 0.161 vs. 1.060 ± 0.092 × 10^−3^ mm^2^/s, *p* < 0.001, d = −1.07). Posterior WM f was also lower in RR-MS patients (0.050 ± 0.072 vs. 0.061 ± 0.012, *p* < 0.001), although the effect size was small (d = −0.17).

In the splenium, RR-MS patients showed significantly higher ADC (0.859 ± 0.147 vs. 0.804 ± 0.061 × 10^−3^ mm^2^/s, *p* = 0.029, d = 0.39) and D (0.833 ± 0.137 vs. 0.768 ± 0.064 × 10^−3^ mm^2^/s, *p* = 0.007, d = 0.50), together with significantly lower D* (0.854 ± 0.158 vs. 0.950 ± 0.131 × 10^−3^ mm^2^/s, *p* < 0.001, d = −0.62). Splenium f did not differ significantly between groups (0.058 ± 0.051 vs. 0.051 ± 0.022, *p* = 0.735).

In the genu, D* was significantly lower in RR-MS patients than in controls (0.849 ± 0.184 vs. 0.917 ± 0.099 × 10^−3^ mm^2^/s, *p* = 0.003, d = −0.39). ADC showed a trend toward higher values in RR-MS patients but did not reach statistical significance (0.848 ± 0.173 vs. 0.795 ± 0.046 × 10^−3^ mm^2^/s, *p* = 0.064), whereas D and f were not significantly different between groups. No significant group differences were observed in frontal WM for any IVIM-DWI parameter (all *p* > 0.05).

Age- and sex-adjusted sensitivity analyses were performed to assess whether the main regional findings persisted after accounting for demographic imbalance between RR-MS patients and healthy controls. Posterior WM differences remained significant for ADC (β = −0.095, FDR-adjusted *p* < 0.001), D (β = −0.082, FDR-adjusted *p* = 0.002), and D* (β = −0.164, FDR-adjusted *p* < 0.001), whereas posterior WM f was not significant after adjustment. In the splenium, D* remained significant after adjustment (β = −0.103, FDR-adjusted *p* = 0.017), whereas the ADC and D differences were attenuated. No significant adjusted group differences were observed in frontal WM. Full adjusted estimates are provided in Supplementary Table S1.

**Figure 3:**
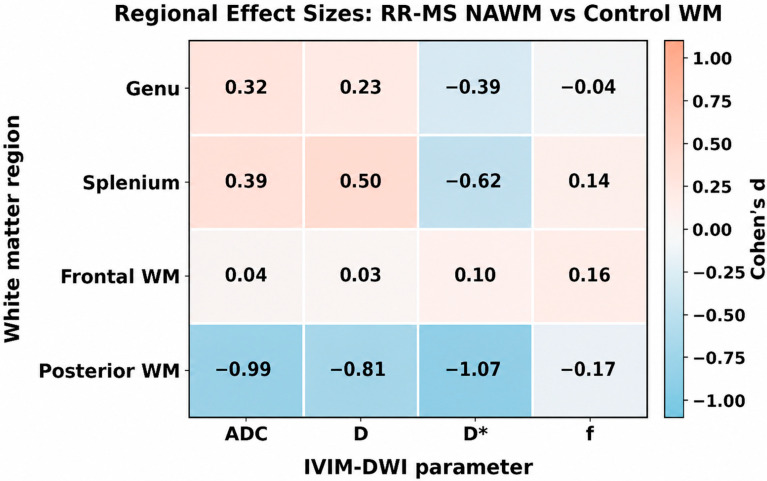
Regional effect-size heatmap of IVIM-DWI differences between RR-MS NAWM and healthy control WM. Cohen’s d values are shown for ADC, D, D*, and f across four WM regions. Negative values indicate lower values in RR-MS NAWM, and positive values indicate higher values in RR-MS NAWM. The strongest unadjusted effects were observed in posterior WM and the splenium. ADC, D, and D* are expressed in ×10^−3^ mm^2^/s.

Overall, the regional analysis demonstrated a non-uniform NAWM abnormality pattern, with the most robust differences localized to posterior WM, followed by a more selective splenial D* abnormality after adjustment. In contrast, no clear IVIM-DWI abnormality was detected in frontal WM across the sampled parameters. The unadjusted regional effect-size pattern is visually summarized in Figure 3.

### 3.3. Contextual Tissue-Compartment IVIM-DWI Analysis

IVIM-DWI parameters were compared across three tissue compartments: averaged control WM, averaged MS NAWM, and lesion-averaged MS tissue (Table 3). For ADC, values were similar between averaged control WM and averaged MS NAWM (0.808 ± 0.030 vs. 0.812 ± 0.076 × 10^−3^ mm^2^/s, *p* = 0.950), whereas lesion-averaged MS tissue showed significantly higher ADC than both averaged MS NAWM and averaged control WM (1.097 ± 0.144 × 10^−3^ mm^2^/s, both *p* < 0.001).

A similar pattern was observed for D, with no significant difference between averaged control WM and averaged MS NAWM (0.769 ± 0.031 vs. 0.776 ± 0.077 × 10^−3^ mm^2^/s, *p* = 0.831), while lesion-averaged MS tissue showed significantly higher D than both comparison compartments (1.043 ± 0.140 × 10^−3^ mm^2^/s, both *p* < 0.001).

For D*, averaged MS NAWM showed lower values than averaged control WM (0.883 ± 0.091 vs. 0.962 ± 0.057 × 10^−3^ mm^2^/s, *p* < 0.001), whereas lesion-averaged MS tissue showed the highest D* values (1.268 ± 0.160 × 10^−3^ mm^2^/s), with significant differences compared with both averaged MS NAWM and averaged control WM (both *p* < 0.001).

For f, values did not differ significantly between averaged control WM and averaged MS NAWM (0.054 ± 0.012 vs. 0.055 ± 0.053, *p* = 0.303) or between averaged control WM and lesion-averaged MS tissue (0.059 ± 0.016, *p* = 0.080). However, lesion-averaged MS tissue showed significantly higher f than averaged MS NAWM (*p* < 0.001).

Overall, lesion-averaged MS tissue demonstrated a distinct IVIM-DWI profile, particularly with higher ADC, D, and D*, whereas averaged MS NAWM was closer to averaged control WM for ADC and D.

## 4. Discussion

This study evaluated whether IVIM-DWI can detect quantitative WM abnormalities in RR-MS beyond conventional MRI appearance and whether these abnormalities differ across anatomically selected WM regions and tissue compartments. The main finding was that IVIM-DWI identified measurable abnormalities in MS NAWM, but these abnormalities were region-specific rather than uniformly distributed. The biggest differences were observed in posterior WM, followed by the splenium, whereas no clear IVIM-DWI abnormality was detected in the sampled frontal WM regions. Age- and sex-adjusted sensitivity analysis supported the robustness of the posterior WM ADC, D, and D* findings, whereas splenial ADC and D differences were attenuated after adjustment and should therefore be interpreted more cautiously. In addition, lesion-averaged MS tissue demonstrated a distinct IVIM-DWI profile, particularly with higher ADC, D, and D*, while averaged MS NAWM was closer to averaged control WM for ADC and D. These findings support IVIM-DWI as an exploratory quantitative tool for regional WM assessment in RR-MS.

The selected regions were chosen to provide focused anatomical sampling of NAWM rather than whole-brain tract mapping. The genu and splenium were included to sample anterior and posterior callosal WM, as the corpus callosum is a major interhemispheric WM pathway frequently affected in MS. Prior DTI work has demonstrated abnormal diffusion characteristics in normal-appearing corpus callosum even after visible lesions are excluded, supporting the presence of occult callosal injury [30]. Additional corpus callosum studies have shown that callosal abnormalities in MS may differ across callosal segments, including anterior and posterior callosal regions, supporting the rationale for sampling both the genu and splenium rather than treating the corpus callosum as a single uniform structure [31,32]. Posterior WM was included because periventricular and deep WM regions are characteristic sites of MS involvement, and previous work has shown that NAWM abnormalities may vary spatially and may relate to regions where new lesions subsequently develop [5,7,13]. The inclusion of frontal and posterior WM was further supported by regional and tract-based diffusion MRI studies showing that NAWM abnormalities may vary across lobar and posterior WM pathways, including frontal, parietal, occipital, callosal, and optic radiation regions [12,33]. Frontal WM was included as an additional non-callosal supratentorial WM region for comparison. Together, these four regions allowed assessment of whether IVIM-DWI abnormalities were broadly distributed or concentrated in specific NAWM territories.

The regional NAWM findings are consistent with prior regional and quantitative MRI studies showing that tissue appearing normal on conventional MRI may still contain occult MS-related abnormalities. Bao et al. used DTI in RR-MS and reported microstructural abnormalities across atlas-defined NAWM regions, supporting the value of regional rather than purely global WM assessment [12]. Similarly, Zhu et al. used diffusion kurtosis imaging (DKI) to evaluate heterogeneous WM areas in RR-MS and showed that advanced diffusion metrics can detect spatially variable microstructural damage in non-lesional WM [20]. Bontempi et al. further demonstrated that multicomponent T2 relaxation can identify abnormalities in non-lesional WM in RR-MS, reinforcing the broader concept that quantitative MRI can reveal tissue changes missed by conventional sequences [19]. In this context, the present study extends prior regional quantitative MRI work by showing that IVIM-DWI can also identify region-dependent NAWM abnormalities, particularly in posterior WM and the splenium.

The posterior WM finding was the most prominent regional result. RR-MS patients showed lower posterior WM ADC, D, D*, and f compared with healthy controls, with large effect sizes for ADC, D, and D*. This pattern differed from the lesion-averaged tissue profile, in which ADC, D, and D* were higher. Therefore, posterior NAWM should not simply be interpreted as a mild form of established lesion tissue; rather, it may represent a distinct non-lesional tissue state detectable by IVIM-DWI. This interpretation is supported by diffusion MRI studies showing that abnormalities may differ across lesions, perilesional WM, and NAWM rather than following a single uniform pattern [34,35,36]. The direction and magnitude of the posterior WM findings therefore support the importance of regional NAWM assessment when evaluating subtle WM involvement in RR-MS.

The splenium was the second key region, showing higher ADC and D but lower D* in RR-MS patients compared with controls. However, after age and sex adjustment, the splenial ADC and D differences were attenuated, whereas the D* difference persisted, supporting a more cautious interpretation of splenial diffusion-related findings. This supports the relevance of callosal WM involvement in MS and suggests that callosal abnormalities may differ by subregion and IVIM-DWI parameter. Prior DTI work has demonstrated abnormal diffusion characteristics in normal-appearing corpus callosum in MS, suggesting occult callosal injury even when visible plaques are excluded [30]. Regional diffusion studies also support location-dependent NAWM abnormalities in RR-MS [12,20,29,30,33]. The segmental callosal literature further supports this interpretation, as prior studies have reported that diffusion abnormalities in MS may differ across callosal subdivisions, including anterior and posterior callosal segments [31,32]. In the present study, the splenium showed a more selective adjusted abnormality pattern than the genu, particularly for D*, suggesting that callosal NAWM involvement is not uniform and may depend on both anatomical location and the quantitative parameter being measured.

The tissue-compartment analysis further supports the utility of IVIM-DWI for distinguishing different WM tissue states. Averaged MS NAWM was close to the averaged control WM for ADC and D, whereas lesion-averaged MS tissue showed clearly higher ADC, D, and D*. This pattern agrees with prior IVIM-DWI studies reporting increased diffusion-related parameters within MS lesions, consistent with overt tissue disruption in demyelinating plaques [25,26,27]. At the same time, the similarity between averaged MS NAWM and averaged control WM for ADC and D highlights the importance of regional analysis. When NAWM is treated as a single averaged compartment, localized abnormalities, particularly in posterior WM and the more selective splenial D* pattern, may be attenuated or missed. Therefore, the combined regional and tissue-compartment findings suggest that IVIM-DWI may capture both subtle non-lesional WM abnormalities and more pronounced changes within established MS lesions.

The D* findings may provide additional insight into differences between non-lesional WM and overt MS lesion tissue. D* is generally considered the IVIM parameter most closely related to pseudo-diffusion from incoherent microvascular motion [21,22,23]. In the present study, D* was lower in several NAWM regions in RR-MS patients compared with controls, and was also lower in averaged MS NAWM than in averaged control WM. This reduction may reflect altered microvascular perfusion or reduced incoherent microcirculatory motion within non-lesional WM, consistent with the prior perfusion MRI literature reporting perfusion abnormalities in MS NAWM [24]. In contrast, lesion-averaged MS tissue showed the highest D* values, suggesting a different pathological environment within overt plaques. This may be related to inflammatory vascular changes, altered microvascular flow, and blood–brain barrier disruption, which have been described during new MS lesion formation [37]. This interpretation is also consistent with prior IVIM-DWI studies showing distinct diffusion- and perfusion-related profiles in MS lesions [25,26,27]. Therefore, the divergent D* pattern between NAWM and lesions supports interpreting IVIM-DWI as a multiparametric technique that captures complementary diffusion-related and perfusion-sensitive aspects of WM pathology.

The main implication of this study is that regional IVIM-DWI may provide an exploratory quantitative approach for WM assessment in RR-MS. Conventional MRI remains essential for detecting visible lesions, but it does not fully characterize non-lesional WM. Regional IVIM-DWI analysis may help identify subtle NAWM abnormalities that are missed by global WM averaging and may serve as a foundation for future studies integrating IVIM-DWI with myelin-sensitive imaging, volumetric MRI, DTI, DKI, relaxation-time measurements, and longitudinal lesion-evolution analysis.

Several limitations should be acknowledged. This was a retrospective cross-sectional study; therefore, the temporal relationship between regional NAWM abnormalities and future lesion development or clinical progression could not be assessed. Potential sources of bias include selection bias related to the retrospective complete-case sampling strategy, measurement bias related to manual ROI placement, and confounding related to the unmatched age and sex distributions between RR-MS patients and healthy controls. Because the RR-MS cohort was retrospectively identified from clinical and imaging records acquired between 2019 and 2024, diagnoses were based on the 2017 McDonald criteria used in clinical practice during the study period. Patients were not reclassified according to the more recent 2024 McDonald criteria [38], published in 2025, which incorporate updated diagnostic considerations and additional MRI-related features. This should be considered when interpreting the diagnostic framework of the cohort. The healthy control group was smaller than the RR-MS group and was not age- or sex-matched to the RR-MS cohort. Although age- and sex-adjusted sensitivity analyses were performed and supported the robustness of the main posterior WM findings, residual confounding may remain because of the limited and demographically imbalanced control cohort. ROIs were carefully placed to avoid visible lesions, CSF spaces, vascular structures, and imaging artifacts; however, ROI-based measurements remain dependent on anatomical sampling and may not fully capture the spatial heterogeneity of NAWM abnormalities. Interobserver reproducibility was not formally assessed, and future studies should evaluate the reproducibility of regional IVIM-DWI ROI measurements using multiple raters or automated atlas-based approaches. Because this study used an exploratory regional design, the findings should be interpreted in relation to both statistical significance and effect-size patterns and should be validated in larger independent cohorts.

Future longitudinal studies should validate these findings and determine whether regional IVIM-DWI abnormalities predict subsequent lesion formation, brain volume loss, or clinical disability progression. Such studies should also examine the relationship between regional NAWM IVIM-DWI abnormalities and total lesion burden, lesion location, and lesion proximity using lesion maps or atlas-based approaches. Future work should further expand anatomical coverage beyond the selected WM regions examined in this study to include additional WM territories, cortical and deep gray matter regions, and whole-brain or atlas-based analyses. Integrating IVIM-DWI with complementary quantitative MRI techniques, including relaxation-time mapping, myelin-sensitive metrics, DTI, DKI, and volumetric MRI, may further clarify how IVIM-derived parameters relate to tissue microstructure, myelin content, and neurodegeneration.

## 5. Conclusions

In this cohort, IVIM-DWI detected region-specific abnormalities in NAWM in patients with RR-MS, most robustly in posterior WM, with a more selective splenial D* abnormality after age and sex adjustment. These findings indicate that NAWM abnormalities are not uniformly distributed and may be missed when WM is analyzed only as a globally averaged compartment. The tissue-compartment analysis further showed that lesion-averaged MS tissue has a distinct IVIM-DWI profile, particularly with higher ADC, D, and D*, compared with averaged MS NAWM and control WM. Overall, regional IVIM-DWI may provide an exploratory quantitative framework for evaluating subtle non-lesional WM signal abnormalities and distinguishing NAWM patterns from established MS lesion tissue. Future longitudinal studies with broader anatomical coverage are needed to validate these findings and determine their relevance to lesion evolution, brain volume loss, and clinical disability progression.

## Figures and Tables

**Table 1:** Participant characteristics of patients with RR-MS and healthy controls.

Variable	RR-MS Patients (*n* = 189)	Healthy Controls (*n* = 26)
Age, years	36.02 ± 9.31	31.73 ± 9.23
Male sex, *n* (%)	57 (30.2%)	23 (88.5%)
Female sex, *n* (%)	132 (69.8%)	3 (11.5%)
Disease duration, years	6.27 ± 5.18	N/A
EDSS	2.25 ± 1.94	N/A
Lesion count	11.57 ± 8.19	N/A
On DMT, *n* (%)	138 (73.0%)	N/A
Not on DMT, *n* (%)	51 (27.0%)	N/A

Data are presented as mean ± SD or *n* (%). Disease duration was available for 185 RR-MS patients. RR-MS = relapsing–remitting multiple sclerosis; EDSS = Expanded Disability Status Scale; DMT = disease-modifying treatment; SD = standard deviation; N/A = not applicable.

**Table 2:** Regional NAWM IVIM-DWI differences between RR-MS patients and healthy controls.

Region	Parameter	RR-MS NAWM	Control WM	*p*-Value	Cohen’s d
**Genu**	**ADC**	0.848 ± 0.173	0.795 ± 0.046	0.064	0.32
	**D**	0.805 ± 0.172	0.768 ± 0.057	0.217	0.23
	D*	0.849 ± 0.184	0.917 ± 0.099	0.003	−0.39
	**f**	0.056 ± 0.059	0.058 ± 0.027	0.351	−0.04
**Splenium**	**ADC**	0.859 ± 0.147	0.804 ± 0.061	0.029	0.39
	**D**	0.833 ± 0.137	0.768 ± 0.064	0.007	0.50
	D*	0.854 ± 0.158	0.950 ± 0.131	<0.001	−0.62
	**f**	0.058 ± 0.051	0.051 ± 0.022	0.735	0.14
**Frontal WM**	**ADC**	0.774 ± 0.078	0.771 ± 0.039	0.417	0.04
	**D**	0.733 ± 0.096	0.731± 0.032	0.518	0.03
	D*	0.933 ± 0.136	0.920± 0.085	0.287	0.10
	**f**	0.055 ± 0.060	0.046± 0.009	0.390	0.16
**Posterior WM**	**ADC**	0.766 ± 0.102	0.862± 0.054	<0.001	−0.99
	**D**	0.729 ± 0.104	0.810± 0.050	<0.001	−0.81
	D*	0.894 ± 0.161	1.060± 0.092	<0.001	−1.07
	**f**	0.050 ± 0.072	0.061± 0.012	<0.001	−0.17

Data are presented as mean ± SD. Between-group comparisons were performed using the Mann–Whitney U test. Cohen’s d reflects the direction and magnitude of the difference between RR-MS NAWM and control WM. ADC, D, and D* are expressed in ×10^−3^ mm^2^/s. ADC = apparent diffusion coefficient; D = true diffusion coefficient; D* = pseudo-diffusion coefficient; f = perfusion fraction; NAWM = normal-appearing white matter; RR-MS = relapsing–remitting multiple sclerosis; WM = white matter.

**Table 3:** Contextual tissue-compartment IVIM-DWI profiles across averaged control WM, averaged MS NAWM, and lesion-averaged MS tissue.

Parameter	Averaged Control WM	Averaged MS NAWM	Lesion-Averaged MS Tissue	*p*-Value Control vs. NAWM	*p*-Value NAWM vs. MS Lesions	*p*-Value Control vs. MS Lesions
ADC	0.808 ± 0.030	0.812 ± 0.076	1.097 ± 0.144	0.950	<0.001	<0.001
D	0.769 ± 0.031	0.776 ± 0.077	1.043 ± 0.140	0.831	<0.001	<0.001
D*	0.962 ± 0.057	0.883 ± 0.091	1.268 ± 0.160	<0.001	<0.001	<0.001
f	0.054 ± 0.012	0.055 ± 0.053	0.059 ± 0.016	0.303	<0.001	0.080

Data are presented as mean ± SD. Averaged control WM represents the mean of the genu, splenium, frontal WM, and posterior WM regions in healthy controls. Averaged MS NAWM represents the mean of the same four regions in patients with RR-MS. Lesion-averaged MS tissue represents the mean IVIM-DWI value across MS lesions for each patient. Comparisons involving healthy controls were performed using the Mann–Whitney U test, whereas comparisons between averaged MS NAWM and lesion-averaged MS tissue were performed using the Wilcoxon signed-rank test. ADC, D, and D* are expressed in ×10^−3^ mm^2^/s. ADC = apparent diffusion coefficient; D = true diffusion coefficient; D* = pseudo-diffusion coefficient; f = perfusion fraction; NAWM = normal-appearing white matter; RR-MS = relapsing–remitting multiple sclerosis; WM = white matter.

## Data Availability

The datasets generated and/or analyzed during the current study are available from the corresponding author upon reasonable request, subject to institutional and ethical restrictions.

## Supplementary Materials

The following supporting information can be downloaded at https://www.mdpi.com/article/10.3390/jcm15145493/s1. Figure S1: Participant flow diagram. Flow diagram showing selection of the RR-MS cohort and healthy controls, including eligibility assessment, exclusions, and final analytical sample. Table S1: Age- and sex-adjusted sensitivity analysis of regional IVIM-DWI differences between RR-MS NAWM and control WM.

## Abbreviations

The following abbreviations are used in this manuscript:

**Abbreviation**	**Definition**
ADC	Apparent diffusion coefficient
D	True diffusion coefficient
D*	Pseudo-diffusion coefficient
f	Perfusion fraction
DKI	Diffusion kurtosis imaging
DTI	Diffusion tensor imaging
FLAIR	Fluid-attenuated inversion recovery
IVIM-DWI	Intravoxel incoherent motion diffusion-weighted imaging
MRI	Magnetic resonance imaging
MS	Multiple sclerosis
NAWM	Normal-appearing white matter
ROI	Region of interest
RR-MS	Relapsing–remitting multiple sclerosis
WM	White matter

## References

[1] Lublin F.D., Häring D.A., Ganjgahi H., Ocampo A., Hatami F., Čuklina J., Aarden P., Dahlke F., Arnold D.L., Wiendl H. (2022). How Patients with Multiple Sclerosis Acquire Disability. Brain.

[2] Walton C., King R., Rechtman L., Kaye W., Leray E., Marrie R.A., Robertson N., La Rocca N., Uitdehaag B., van Der Mei I. (2020). Rising Prevalence of Multiple Sclerosis Worldwide: Insights from the Atlas of Ms. Mult. Scler. J..

[3] Mey G.M., Mahajan K.R., DeSilva T.M. (2023). Neurodegeneration in Multiple Sclerosis. WIREs Mech. Dis..

[4] Simkins T.J., Duncan G.J., Bourdette D. (2021). Chronic Demyelination and Axonal Degeneration in Multiple Sclerosis: Pathogenesis and Therapeutic Implications. Curr. Neurol. Neurosci. Rep..

[5] Filippi M., Preziosa P., Banwell B.L., Barkhof F., Ciccarelli O., De Stefano N., Geurts J.J., Paul F., Reich D.S., Toosy A.T. (2019). Assessment of Lesions on Magnetic Resonance Imaging in Multiple Sclerosis: Practical Guidelines. Brain.

[6] Thompson A.J., Banwell B.L., Barkhof F., Carroll W.M., Coetzee T., Comi G., Correale J., Fazekas F., Filippi M., Freedman M.S. (2018). Diagnosis of Multiple Sclerosis: 2017 Revisions of the Mcdonald Criteria. Lancet Neurol..

[7] Wattjes M.P., Ciccarelli O., Reich D.S., Banwell B., De Stefano N., Enzinger C., Fazekas F., Filippi M., Frederiksen J., Gasperini C. (2021). 2021 Magnims–Cmsc–Naims Consensus Recommendations on the Use of Mri in Patients with Multiple Sclerosis. Lancet Neurol..

[8] Filippi M., Preziosa P., Arnold D.L., Barkhof F., Harrison D.M., Maggi P., Mainero C., Montalban X., Sechi E., Weinshenker B.G. (2023). Present and Future of the Diagnostic Work-up of Multiple Sclerosis: The Imaging Perspective. J. Neurol..

[9] Barkhof F. (2002). The Clinico-Radiological Paradox in Multiple Sclerosis Revisited. Curr. Opin. Neurol..

[10] Dünschede J., Ruschil C., Bender B., Mengel A., Lindig T., Ziemann U., Kowarik M.C. (2023). Clinical-Radiological Mismatch in Multiple Sclerosis Patients During Acute Relapse: Discrepancy between Clinical Symptoms and Active, Topographically Fitting Mri Lesions. J. Clin. Med..

[11] Elahi R., Taremi S., Najafi A., Karimi H., Asadollahzadeh E., Sajedi S.A., Rad H.S., Sahraian M.A. (2025). Advanced Mri Methods for Diagnosis and Monitoring of Multiple Sclerosis (Ms). J. Magn. Reson. Imaging.

[12] Bao J., Tu H., Li Y., Sun J., Hu Z., Zhang F., Li J. (2022). Diffusion Tensor Imaging Revealed Microstructural Changes in Normal-Appearing White Matter Regions in Relapsing–Remitting Multiple Sclerosis. Front. Neurosci..

[13] Elliott C., Momayyezsiahkal P., Arnold D.L., Liu D., Ke J., Zhu L., Zhu B., George I.C., Bradley D.P., Fisher E. (2021). Abnormalities in Normal-Appearing White Matter from Which Multiple Sclerosis Lesions Arise. Brain Commun..

[14] Larassati H., Pandelaki J., Estiasari R., Prihartono J., Firdausia S., Yunus R.E., Mulyadi R. (2022). Diffusion Magnetic Resonance Imaging of Normal-Appearing White Matter in Multiple Sclerosis: Correlation with Brain Volume and Clinical Disability. J. Cent. Nerv. Syst. Dis..

[15] Lopez-Soley E., Martinez-Heras E., Solana E., Solanes A., Radua J., Vivo F., Prados F., Sepúlveda M., Cabrera-Maqueda J.M., Fonseca E. (2023). Diffusion Tensor Imaging Metrics Associated with Future Disability in Multiple Sclerosis. Sci. Rep..

[16] Musall B.C., Gabr R.E., Yang Y., Kamali A., Lincoln J.A., Jacobs M.A., Ly V., Luo X., Wolinsky J.S., Narayana P.A. (2024). Detection of Diffusely Abnormal White Matter in Multiple Sclerosis on Multiparametric Brain Mri Using Semi-Supervised Deep Learning. Sci. Rep..

[17] Muñoz González G., ’t Hart B.A., Bugiani M., Plemel J.R., Schenk G.J., Kooij G., Luchicchi A. (2025). A Focus on the Normal-Appearing White and Gray Matter within the Multiple Sclerosis Brain: A Link to Smoldering Progression. Acta Neuropathol..

[18] Caranova M., Soares J.F., Batista S., Castelo-Branco M., Duarte J.V. (2023). A Systematic Review of Microstructural Abnormalities in Multiple Sclerosis Detected with Noddi and Dti Models of Diffusion-Weighted Magnetic Resonance Imaging. Magn. Reson. Imaging.

[19] Bontempi P., Rozzanigo U., Marangoni S., Fogazzi E., Ravanelli D., Cazzoletti L., Giometto B., Farace P. (2023). Non-Lesional White Matter in Relapsing–Remitting Multiple Sclerosis Assessed by Multicomponent T2 Relaxation. Brain Behav..

[20] Zhu Q., Zheng Q., Luo D., Peng Y., Yan Z., Wang X., Chen X., Li Y. (2022). The Application of Diffusion Kurtosis Imaging on the Heterogeneous White Matter in Relapsing-Remitting Multiple Sclerosis. Front. Neurosci..

[21] Le Bihan D. (2019). What Can We See with Ivim Mri?. Neuroimage.

[22] Le Bihan D., Breton E., Lallemand D., Grenier P., Cabanis E., Laval-Jeantet M. (1986). Mr Imaging of Intravoxel Incoherent Motions: Application to Diffusion and Perfusion in Neurologic Disorders. Radiology.

[23] Paschoal A.M., Leoni R.F., Dos Santos A.C., Paiva F.F. (2018). Intravoxel Incoherent Motion Mri in Neurological and Cerebrovascular Diseases. NeuroImage Clin..

[24] Lapointe E., Li D., Traboulsee A., Rauscher A. (2018). What Have We Learned from Perfusion Mri in Multiple Sclerosis?. Am. J. Neuroradiol..

[25] Alomair O.I., Alghamdi S.A., Abujamea A.H., AlfIfi A.Y., Alashban Y.I., Kurniawan N.D. (2025). Investigating the Role of Intravoxel Incoherent Motion Diffusion-Weighted Imaging in Evaluating Multiple Sclerosis Lesions. Diagnostics.

[26] Alomair O.I., Alghamdi S.A., Abujamea A.H., Aljarallah S., Alkhawajah N.M., Alshuhri M.S., Alashban Y.I., Kurniawan N.D. (2025). The Utility of Intravoxel Incoherent Motion Metrics in Assessing Disability in Relapsing–Remitting Multiple Sclerosis. Diagnostics.

[27] Alomair O.I., Alshuhri M.S., Al-Mubarak H.F., Alghamdi S.A., Abujamea A.H., Aljarallah S., Alkhawajah N.M., Alashban Y.I., Kurniawan N.D. (2025). Ivim-Dwi-Based Radiomics for Lesion Phenotyping and Clinical Status Prediction in Relapsing–Remitting Multiple Sclerosis. J. Clin. Med..

[28] Alghamdi S.A., Alomair O.I., Abujamea A.H. (2026). Multimodal Quantitative Mri of White and Gray Matter: Associations with Myelin-Related Metrics in the Healthy Adult Brain Using Synthetic Mri and Ivim-Dwi. J. Clin. Med..

[29] Yushkevich P.A., Piven J., Hazlett H.C., Smith R.G., Ho S., Gee J.C., Gerig G. (2006). User-Guided 3d Active Contour Segmentation of Anatomical Structures: Significantly Improved Efficiency and Reliability. Neuroimage.

[30] Coombs B.D., Best A., Brown M.S., Miller D.E., Corboy J., Baier M., Simon J.H. (2004). Multiple Sclerosis Pathology in the Normal and Abnormal Appearing White Matter of the Corpus Callosum by Diffusion Tensor Imaging. Mult. Scler. J..

[31] Hasan K.M., Gupta R.K., Santos R.M., Wolinsky J.S., Narayana P.A. (2005). Diffusion Tensor Fractional Anisotropy of the Normal-Appearing Seven Segments of the Corpus Callosum in Healthy Adults and Relapsing-Remitting Multiple Sclerosis Patients. J. Magn. Reson. Imaging Off. J. Int. Soc. Magn. Reson. Med..

[32] Ozturk A., Smith S., Gordon-Lipkin E., Harrison D., Shiee N., Pham D., Caffo B., Calabresi P., Reich D. (2010). Mri of the Corpus Callosum in Multiple Sclerosis: Association with Disability. Mult. Scler. J..

[33] Oladosu O., Liu W.-Q., Brown L., Pike B.G., Metz L.M., Zhang Y. (2022). Advanced Diffusion Mri and Image Texture Analysis Detect Widespread Brain Structural Differences between Relapsing-Remitting and Secondary Progressive Multiple Sclerosis. Front. Hum. Neurosci..

[34] Gloor M., Andelova M., Gaetano L., Papadopoulou A., Burguet Villena F., Sprenger T., Radue E.-W., Kappos L., Bieri O., Garcia M. (2024). Longitudinal Analysis of New Multiple Sclerosis Lesions with Magnetization Transfer and Diffusion Tensor Imaging. Eur. Radiol..

[35] Shi Z., Pan Y., Yan Z., Ding S., Hu H., Wei Y., Luo D., Xu Y., Zhu Q., Li Y. (2023). Microstructural Alterations in Different Types of Lesions and Their Perilesional White Matter in Relapsing-Remitting Multiple Sclerosis Based on Diffusion Kurtosis Imaging. Mult. Scler. Relat. Disord..

[36] van der Weijden C.W., van der Hoorn A., Potze J.H., Renken R.J., Borra R.J., Dierckx R.A., Gutmann I.W., Ouaalam H., Karimi D., Gholipour A. (2022). Diffusion-Derived Parameters in Lesions, Peri-Lesion and Normal-Appearing White Matter in Multiple Sclerosis Using Tensor, Kurtosis and Fixel-Based Analysis. J. Cereb. Blood Flow Metab..

[37] Gaitán M.I., Shea C.D., Evangelou I.E., Stone R.D., Fenton K.M., Bielekova B., Massacesi L., Reich D.S. (2011). Evolution of the Blood–Brain Barrier in Newly Forming Multiple Sclerosis Lesions. Ann. Neurol..

[38] Montalban X., Lebrun-Frénay C., Oh J., Arrambide G., Moccia M., Amato M.P., Amezcua L., Banwell B., Bar-Or A., Barkhof F. (2025). Diagnosis of Multiple Sclerosis: 2024 Revisions of the Mcdonald Criteria. Lancet Neurol..

