# Supplementary material for: Regional IVIM-DWI Abnormalities in Normal-Appearing White Matter and Lesion Tissue in Relapsing–Remitting Multiple Sclerosis
Source: J Clin Med. 2026 Jul 13;15(14):5493. doi: 10.3390/jcm15145493 (PMC13412639; doi:10.3390/jcm15145493)
Supplement: Supplementary file 1 [file jcm-15-05493-s001.zip › jcm-4411696-supplementary.pdf]

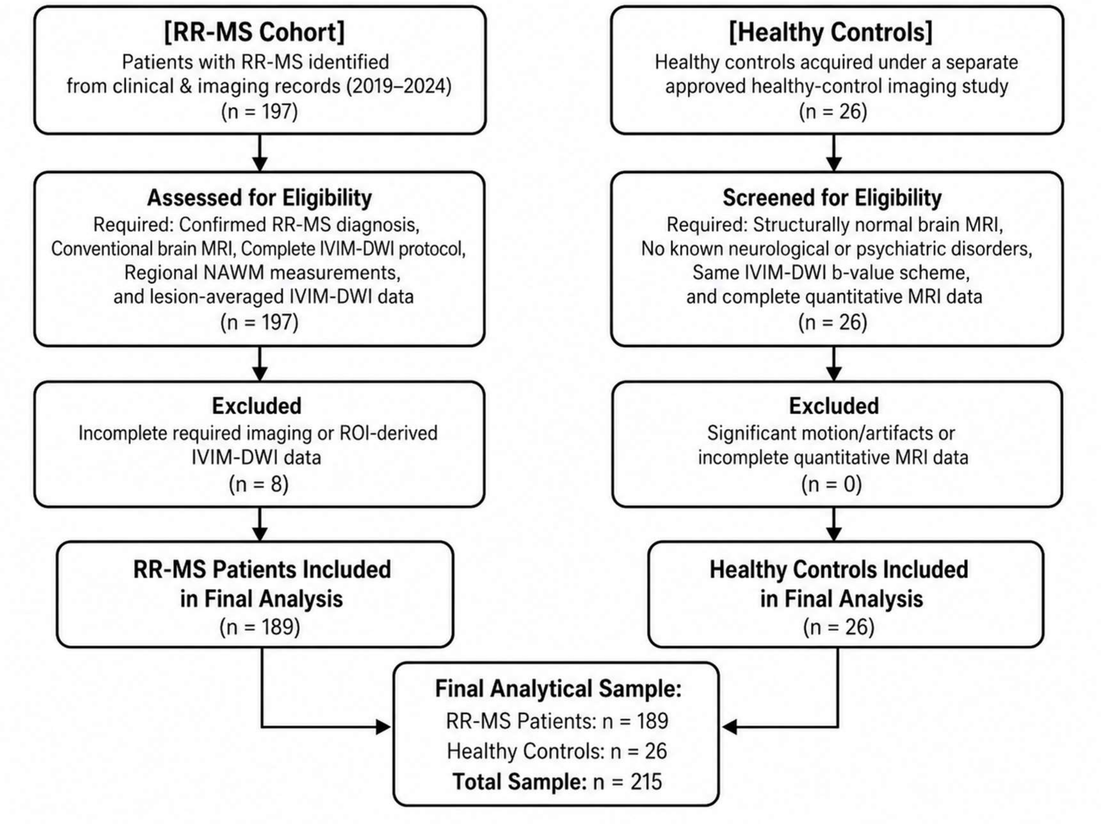

Supplementary Figure S1. Participant flow diagram. Flow diagram showing selection of the RR-MS cohort and healthy controls, including eligibility assessment, exclusions, and final analytical sample.

Table S1. Age- and sex-adjusted sensitivity analysis of regional IVIM-DWI differences between RR-MS NAWM and control WM.

Region	Parameter	Adjusted β, RR-MS vs control	SE	p-value	FDR-adjusted p
Genu	D	0.047	0.037	0.208	0.370
	ADC	0.065	0.037	0.085	0.195
	D*	−0.057	0.040	0.158	0.316
	f	0.006	0.013	0.667	0.711
Splenium	ADC	0.056	0.032	0.085	0.195
	D	0.058	0.030	0.053	0.171
	D*	−0.103	0.036	0.004	0.017
	f	0.009	0.011	0.437	0.543
	ADC	−0.013	0.017	0.441	0.543

**Frontal WM**	**D**	−0.018	0.020	0.387	0.543
	**D***	−0.014	0.030	0.641	0.711
	**f**	0.014	0.013	0.297	0.476
**Posterior WM**	**ADC**	−0.095	0.022	<0.001	<0.001
	**D**	−0.082	0.023	<0.001	0.002
	**D***	−0.164	0.035	<0.001	<0.001
	**f**	−0.002	0.015	0.880	0.880
